# Metabolic syndrome and its components in Polish women of childbearing age: a nationwide study

**DOI:** 10.1186/s12889-017-4564-5

**Published:** 2017-07-13

**Authors:** Dorota Szostak-Węgierek, Anna Waśkiewicz, Walerian Piotrowski, Urszula Stepaniak, Andrzej Pająk, Magdalena Kwaśniewska, Paweł Nadrowski, Arkadiusz Niklas, Aleksandra Puch-Walczak, Wojciech Drygas

**Affiliations:** 1 Department of Human Nutrition, Faculty of Health Sciences, Medical University of Warsaw, Erazma Ciołka 27, 01-445 Warsaw, Poland; 2 Department of Epidemiology, Cardiovascular Disease Prevention and Health Promotion, Institute of Cardiology, Warsaw, Poland; 3 Deparment of Epidemiology and Population Studies, Institute of Public Health, Faculty of Health Sciences, Jagiellonian University Medical College, Crakow, Poland; 4 Department of Social and Preventive Medicine, Medical University of Lodz, Lodz, Poland; 5 3rd Department of Cardiology, Upper Silesian Centre of Cardiology, Medical University of Silesia, Katowice, Poland; 6 Department of Hypertension, Angiology, and Internal Medicine, University of Medical Sciences, Poznan, Poland; 7 Department of Arterial Hypertension and Diabetology, Medical University of Gdansk, Gdansk, Poland

**Keywords:** Polish females of childbearing age, Body mass, Lipid disturbances, Glucose metabolism, Metabolic syndrome

## Abstract

**Background:**

Abnormal body mass and related metabolic disorders may affect female reproductive health. The purpose of the study was to determine the prevalence of underweight, overweight, obesity, lipid and glucose metabolism disorders, hypertension, and metabolic syndrome, among Polish women of childbearing age.

**Methods:**

One thousand five hundred eighty-eight non-pregnant Polish women of childbearing age (20–49 years) who participated in the Multi-Centre National Population Health Examination Survey (WOBASZ II study) in 2013–2014, were assigned to 3 age groups: 20–29 years (*n* = 403), 30–39 years (*n* = 600) and 40–49 years (*n* = 585). Measurements of weight, height, waist circumference, blood pressure, blood lipids, and blood glucose were taken. For statistical analysis, the Kruskal-Wallis, Chi-Square, and Cohran-Armitage tests were used.

**Results:**

Of the participants, 4.3% were determined to be underweight, 25.2% were overweight, 15% were obese, and 53.1% had abdominal obesity. With age, the prevalence of both excessive body mass and abdominal obesity tended to increase, and that of underweight to decrease. Frequency of hypercholesterolemia and hypertriglyceridemia found in the whole group were 50% and 12.6% respectively, and also tended to rise with age. Low serum HDL-cholesterol (high density lipoprotein cholesterol) levels were found in 15.1% of the participants. Prevalence of impaired fasting glucose in the whole group was 8.2% and tended to increase with age. Diabetes was found in 1.2% of the participants and its prevalence also tended to rise with age, at the borderline of significance. Frequency of arterial hypertension and metabolic syndrome in the whole group was 15.7% and 14.1% respectively and both tended to increase with age.

**Conclusions:**

Overweight and obesity, especially of abdominal type, and the related metabolic abnormalities are common in Polish women of childbearing age. Their prevalence tends to increase with age. Underweight is relatively common in the youngest age group.

## Background

Metabolic and hormonal disturbances related to both underweight and excessive body mass may adversely affect female procreative health. It concerns both decreased ability to conceive and complications of pregnancy. Low body mass may lead to ovulatory disorders [1], and to suppressed fetal development resulting in decreased birthweight [2–4]. Overweight and obesity may also cause ovulatory disorders and subfecundity [1, 5]. However, contrary to underweight, excessive body mass is linked to an increased risk of macrosomia [6, 7].

It should be emphasized that maternal overweight and obesity are risk factors for gestational diabetes (GDM) and hypertensive disorders of pregnancy (HDP) [8–10] which often result in disturbed fetal development. Furthermore, a woman’s risk of metabolic syndrome and its components increases with each pregnancy [11, 12].

In an earlier study carried out in 2011–2012, with 164 women of childbearing age living in Warsaw, we showed that excessive body mass and related metabolic disorders were very common and that some of them tended to increase with age, at least at the borderline of significance [13]. This study aimed to determine the rates of abnormal body mass and related metabolic disorders among the various age groups of a representative sample of Polish women of childbearing age.

## Methods

The study group consisted of 1588 non-pregnant women of childbearing age (20–49 years), who in 2013–2014 took part in the Multi-Centre National Population Health Examination Survey (WOBASZ II study). The project was implemented by the Institute of Cardiology, together with five of Poland’s Medical Universities. The study was accepted by the Bioethics Committee of Institute of Cardiology in Warsaw. The details concerning the goals, methods, and organization chart were presented elsewhere [14].

The WOBASZ II survey included a random sample of inhabitants from all over Poland, aged 20 years and above. The randomization had two stages. In each of 16 voivodships, 6 communities were drawn: two small (less than 8000 citizens), two medium (8000–40,000 citizens), and two large (more than 40,000 citizens), plus the voivodship’s capital, provided it had not been sampled as a large community. Thereafter, 70 males and 70 females were drawn from each community. Altogether, 15,120 subjects (including 1557 ineligible persons) were sampled from 108 communities. The sampling frame included all adults permanently registered in the surveyed communities. The personal database of the Ministry of the Interior’s Department of State Registers (an electronic register known as PESEL) was used. A total of 6170 respondents throughout Poland participated in the study, which was 45.5% of the total sample of available randomized subjects. A wide range of classical cardiovascular risk factors among the participants of the WOBASZ II study were estimated, using a questionnaire, laboratory tests, and anthropometric measurements. An earlier WOBASZ survey, performed in 2003–2005, was performed using similar methodology and included 13,545 subjects [15].

In the present study, female subjects were assigned to three age groups as follows: 20–29 years (*n* = 403), 30–39 years (*n* = 600), and 40–49 years (*n* = 585). The analysis included demographic characteristics (marital status, education, occupational activity, and the net household income per person), tobacco use, basic health data (self-assessment of health status, oral contraception use, number of children borne, presence of thyroid disease), body mass, waist circumference, lipid and glucose concentrations, blood pressure and presence of metabolic syndrome. Means, standard deviations, and medians were determined for the Body Mass Index (BMI), waist circumference, and serum concentrations of total cholesterol (TC), LDL-cholesterol (low density lipoprotein cholesterol), HDL-cholesterol and triglycerides (TG), along with systolic and diastolic blood pressure (SBP and DBP respectively). Underweight, overweight and obesity were defined according to WHO (World Health Organization) criteria [16]; excessive waist circumference was defined according to both ATP III (Adult Treatment Panel III) [17] and IDF (International Diabetes Federation) [18] criteria. Hypercholesterolaemia, hypertriglceridaemia (HTG) and low HDL-cholesterol levels were recognized using criteria adopted by the European Society of Cardiology [19]. Low HDL-cholesterol concentrations were also defined according to the IDF criteria [18]. Abnormal fasting glucose levels were recognized according to the recommendations of the Polish Diabetic Society [20], arterial hypertension according to the ESH/ESC (European Society of Cardiology and European Society of Hypertension) recommendations from 2013 [21], and metabolic syndrome (MS) using criteria established by IDF in 2005 [18].

### Laboratory measurements

Laboratory tests were performed in a single location, Diagnostyka Central Laboratory at the Institute of Cardiology in Warsaw, which holds a CDC certificate (the Centre for Disease Control Lipid Standardization Program, Atlanta, USA) and the RIQAS (Random International Quality Assessment Scheme), a European certificate of quality. Measurements of lipid and glucose concentrations were performed by means of the Cobas 6000 analyzer, manufactured by Roche, using agents also purchased from the company. Total cholesterol and triglyceride concentrations were measured using enzymatic-colorimetric methods; LDL- and HDL-cholesterol concentrations by uniform colorimetric enzymatic methods; and glucose concentration by the enzymatic method. The details concerning blood sampling, transport, and methodology of measurements are described elsewhere [14].

### Assessment of hypertension

The blood pressure measurement was performed three times on the right arm by means of automatic AND UA-631 devices, which are approved by AAMI (Association for the Advancement of Medical Instrumentation). To assess blood pressure value, the average from the second and third measurements was taken. Hypertension was defined as systolic blood pressure ≥ 140 mmHg and/or diastolic blood pressure ≥ 90 mmHg, or if a subject reported antihypertensive drug treatment.

### Statistical analysis

Continuous variables are presented as means ± standard deviations and medians. Categorical variables are shown as frequencies and percentages (%). Significant differences between age groups were determined by the Kruskal-Wallis and Chi-Square tests. The significance of trends was assessed by the Cohran-Armitage test. A two-sided *p*-value <0.05 was considered statistically significant. Statistical analyses were carried out using the SAS for Windows software package, ver. 9.2.

## Results

Table 1 presents data on marital status, education, occupational activity, net household income per person, and also the basic health data of the subjects under study in the three age groups. Older participants were more frequently married and occupationally active than the younger subjects. On the contrary, the younger females were better educated. Older women declared good or very good health status less frequently and suffered because of diseases of the thyroid gland more often than their younger counterparts.

**Table 1:** Basic characteristics of the women under study

Parameter	All *n* = 1588	Age (years)			*P* value
		20–29 *n* = 403	30–39 *n* = 600	40–49 *n* = 585	
Marital status:					
Married (%)	68.3	35.7	79.8	79.0	<0.0001
Education:					
Primary (%)	19.8	3.7	22.7	27.9	<0.0001
Secondary (%)	38.7	43.9	32.2	41.8	
Higher (%)	41.5	52.4	45.1	30.3	
Occupational activity (%)					
Yes	64.0	52.1	65.9	70.1	<0.0001
No	36.0	47.9	34.1	29.9	
Net household income p.p. (%)^a^					
≤ 500 PLN	16.5	15.9	17.9	15.4	ns
501–1000 PLN	33.5	31.4	33.0	35.3	
1001–2000 PLN	36.9	37.8	36.2	37.0	
2001–3000 PLN	10.1	12.0	9.2	9.9	
> 3000 PLN	3.0	2.9	3.7	2.4	
Smokers (%)^b^	19.3	17.9	17.4	22.2	ns
Self-assessment of health status (%)					
Very good + good	85.7	92.5	91.0	75.6	<0.0001
Average	13.4	7.5	8.7	22.3	
Bad + very bad	0.9	0.0	0.3	2.1	
Oral contraception (%)	12.7	13.9	16.0	8.0	0.0003
Number of born children					
x ± SD	1.51 ± 1.21	0.46 ± 0.73	1.63 ± 1.02	2.07 ± 1.20	
﻿﻿Median	2.00	0	2.00	2.00	0.0001
Number of born children					
0 (%)	24.01	65.62	13.32	7.36	0.0001
1 (%)	24.85	24.15	28.84	21.19	
2 (%)	35.86	8.66	45.19	44.31	
3 (%)	10.16	1.31	8.94	17.34	
4 and more	5.11	0.26	3.71	9.81	
Diseases of the thyroid gland (%)	12.9	9.0	11.5	17.1	0.0005

^a^included only those who answered the question, i.e. 136 subjects

^b^at least one cigarette per day currently

**Fig. 1:**
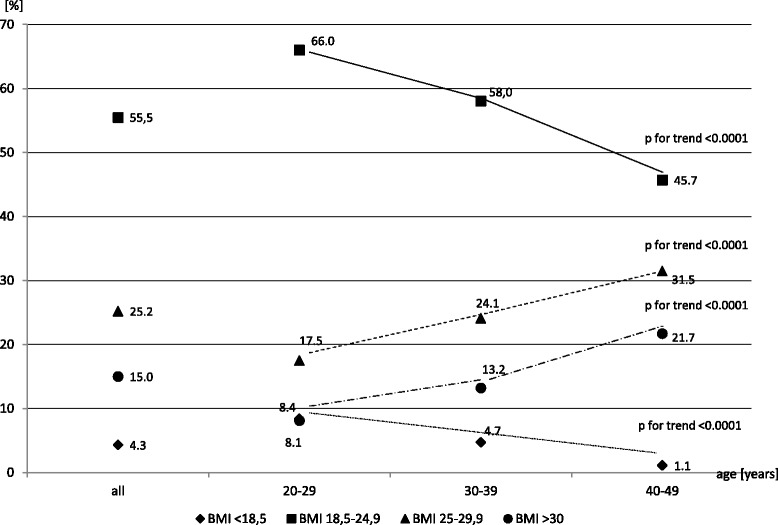
Percentages of women with different BMI ranges across age groups

Mean BMI, waist circumference values, and percentages of women with abnormal body mass and high waist circumference are shown in Table 2. The first two variables tended to increase significantly with age. Similarly, rates of overweight, obesity, and abdominal obesity exhibited a significant trend of rising with age. On the contrary, rates of underweight (BMI < 18.5 kg/m^2^) tended to decrease with age (Fig. 1).

**Table 2:** Anthropometric characteristics in different age groups

Parameter	All	Age (years)			*P* value	*P* for trend
		20–29	30–39	40–49		
BMI (kg/m^2^)	*n* = 1518					
x ± SD	24.9 ± 5.2	22.9 ± 4.1	24.7 ± 4.9	26.5 ± 5.6	<0.0001	<0.0001
Median	23.8	22.0	23.6	25.3		
BMI (%)						
<18.5 kg/m^2^	4.3	8.4	4.7	1.1	<0.0001	<0.0001
18.5–24.9 kg/m^2^	55.5	66.0	58.0	45.7		<0.0001
25–29.9 kg/m^2^	25.2	17.5	24.1	31.5		<0.0001
≥30 kg/m^2^	15.0	8.1	13.2	21.7		<0.0001
Waist (cm)	*N* = 1564					
x ± SD	82.2 ± 12.1	77.3 ± 11.2	82.4 ± 12.1	85.3 ± 11.6	<0.0001	<0.0001
Median	80.0	75.3	80.0	83.0		
Waist (%)^a^						
<80 cm	46.9	65.0	46.6	34.8		
≥ 80 cm	53.1	35.0	53.4	65.2	<0.0001	<0.0001
Waist (%)^b^						
<88 cm	70.4	82.7	70.2	62.3		
≥ 88 cm	29.6	17.3	29.8	37.7	<0.0001	<0.0001

^a^according to the IDF criteria, ^b^according to the ATP III criteria

Lipid profile data and rates of lipid abnormalities are shown in Table 3. Total cholesterol, LDL-cholesterol, and triglyceride concentrations showed a significant tendency to rise with age. However, there were no significant differences in HDL-cholesterol level in the age groups. Prevalence of hypercholesterolemia (defined both as elevated total and as LDL-cholesterol) and hypertriglyceridemia tended to increase significantly with age. There were no significant differences in the rates of low HDL-cholesterol levels in the age groups. A tendency to rise with age, at the level of borderline significance, was only found when low HDL-cholesterol levels were defined according to IDF criteria.

**Table 3:** Serum lipid characteristics in different age groups

Parameter	All	Age (years)			*P* value	*P* for trend
		20–29	30–39	40–49		
TC (mmol/l)	*N* = 1528					
x ± SD	4.90 ± 1.05	4.54 ± 0.97	4.81 ± 1.03	5.23 ± 1.02	<0.0001	<0.0001
Median	4.89	4.51	4.77	5.24		
LDL-chol (mmol/l)	*N* = 1528					
x ± SD	2.85 ± 0.85	2.53 ± 0.77	2.79 ± 0.82	3.15 ± 0.85	<0.0001	<0.0001
Median	2.78	2.46	2.69	3.13		
TG (mmol/l)	*N* = 1527					
x ± SD	1.14 ± 0.70	0.99 ± 0.43	1.11 ± 0.79	1.27 ± 0.73	<0.0001	<0.0001
Median	0.98	0.89	0.95	1.09		
HDL-chol (mmol/l)	*N* = 1528					
x ± SD	1.58 ± 0.42	1.60 ± 0.42	1.58 ± 0.42	1.55 ± 0.41	ns	
Median	1.55	1.59	1.54	1.54		
TC (%)						
TC < 4.9 mmol/l	50.0	65.7	55.6	33.6		
TC ≥ 4.9 mmol/l^a^	50.0	34.3	44.4	66.4	<0.0001	<0.0001
LDL-chol (%)						
LDL-chol <3.00 mmol/l	59.4	77.3	63.9	42.5		
LDL-chol ≥3.00 mmol/l^a^	40.6	22.7	36.1	57.5	<0.0001	<0.0001
TG (%)						
< 1.7 mmol/l	87.4	93.8	89.9	80.3		
≥ 1.7 mmol/l	12.6	6.2	10.1	19.7	<0.0001	<0.0001
HDL-chol (%)^b^						
HDL-chol <1.16 mmol/l	15.1	14.4	14.4	16.3	ns	
HDL-chol ≥1.16 mmol/l	84.9	85.6	85.6	83.7		
HDL-chol (%)^c^						
HDL-chol <1.3 mmol/l	25.4	23.4	23.4	28.8	0.0697	
HDL-chol ≥1.3 mmol/l	74.6	76.6	76.6	71.2		

^a^or hypolipemic medication during 3 preceding days.

^b^according to the criteria of the European Society of Cardiology, c according to the IDF criteria

**Fig. 2:**
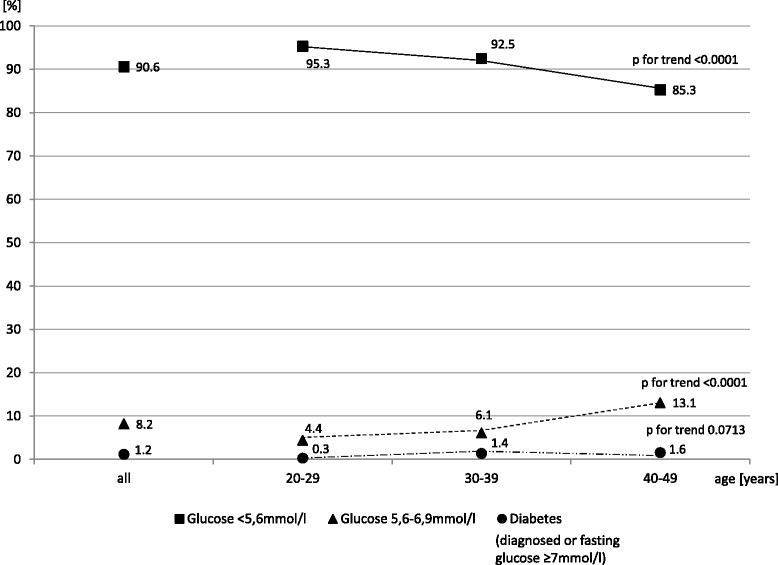
Percentages of women with different serum glucose levels categories across age groups

Rates of glucose metabolism disorders, arterial hypertension, and MS are presented in Table 4. Both fasting glucose concentrations and the prevalence of impaired fasting glucose levels (IFG) tended to rise significantly with age. Prevalence of diabetes also exhibited a tendency to rise with age, but without reaching statistical significance (Fig. 2). Both systolic and diastolic blood pressure values and arterial hypertension rates showed a significant likelihood to increase with age. The prevalence of MS tended to rise significantly with age as well.

**Table 4:** Glucose metabolism and blood pressure characteristics, and metabolic syndrome prevalence in different age groups

Parameter	All	Age (years)			*P* value	*P* for trend
		20–29	30–39	40–49		
Fasting glucose (mmol/l)	*N* = 1529					
x ± SD	4.99 ± 0.75	4.82 ± 0.62	4.99 ± 0.89	5.12 ± 0,63	<0.0001	<0.0001
Median	4.93	4.77	4.91	5.07		
Fasting glucose (%)						
Normal (<5.6 mmol/l)	90.6	95.3	92.5	85.3		<0.0001
IFG (5,6–6,9 mmol/l)	8.2	4.4	6.1	13.1	<0.0001	<0.0001
Diabetes (diagnosed or fasting glucose ≥7 mmol/l)	1.2	0.3	1.4	1.6		0.0713
SBP (mm Hg)	*N* = 1566					
x ± SD	118.4 ± 13.6	114.7 ± 10.3	116.4 ± 12.6	123.0 ± 15.2	<0.0001	<0.0001
Median	117.0	114.5	115.0	121.0		
DBP (mm Hg)						
x ± SD	77.0 ± 10.1	74.0 ± 8.6	76.2 ± 9.8	79.8 ± 10.7	<0.0001	<0.0001
Median	76.5	74.0	76.0	79.0		
BP (%)						
< 140/90 mmHg	84.3	96.3	87.5	72.8		
≥ 140/90 mmHg or medication	15.7	3.7	12.5	27.2	<0.0001	<0.0001
MS (%) according to IDF						
Yes	14.1	5.3	10.4	23.8	<0.0001	<0.0001
No	85.9	94.7	89.6	76.2		

IFG- impaired fasting glucose

SBP- systolic blood pressure; DBP- diastolic blood pressure; BP- blood pressure

## Discussion

In the representative sample of Polish women of childbearing age, the prevalence of overweight and obesity, of abdominal type, and of most of the related metabolic abnormalities tended to increase significantly with age. Abnormal body mass was found in more than 37% of the participants in their thirties and more than 53% of those who were at least 40 years old. Abdominal obesity, recognized according to the IDF criteria, was present in every third woman in the youngest age group and almost in two thirds of women in the oldest. Metabolic syndrome occurred in almost every fourth female in the oldest group. This could be related not only to age itself, but also to the larger number of pregnancies that occurred in this group (Table 1) and pregnancy-related complications, such as GDM and HDP. However, we do not have data about the prevalence of these disorders, which is a limitation of our study. Meanwhile, underweight occurred in over 8% of the youngest participants, and its prevalence tended to decrease with age.

These results are similar to that of our previous study conducted in females of childbearing age living in Warsaw in 2011–2012 [13]. However, abdominal obesity in Warsaw inhabitants was less frequent and the increasing trends of prevalence of metabolic abnormalities with age were not significant. Only the trends for low HDL-levels and MS reached the borderline of significance. We presume that it resulted from a relatively small size of the sample under study (*n* = 164).

The overall mean BMI value in the current study was 24.9 ± 5.2 and increased with age, starting at 22.9 kg/m^2^ in the youngest group and rising to 26.5 kg/m^2^ in the oldest. The previous WOBASZ study [15], conducted in 2003–2005, showed similar results in females aged 20–44 years. However, in the present study, the overall overweight and obesity rates were much higher, 25.2% vs 19%, and 15% vs 9.6% respectively. Underweight, by contrast, occurred in women of childbearing age in the same percentage of participants (about 4%) in both of the WOBASZ studies. Excessive body mass is thus unequivocally more frequent in Polish women of childbearing age than underweight and its prevalence has increased in the last decade.

When compared to American women of childbearing age who took part in the National Health and Nutrition Examination Survey (NHANES), conducted from 1999 to 2008, the participants of our study were much leaner. The mean BMI of US women aged 16–49 years was 27.7 kg/m^2^ [22].

Lipid disorders were common in the participants of our study. Their prevalence was somewhat higher than a decade ago in females aged 20–44 years. Frequencies of hypercholesterolemia (defined as LDL-cholesterol ≥3.0 mmol/l), hypertriglyceridemia and low HDL-chol levels (according to IDF criteria) were 40.6% vs 37%, 12.6% vs 10.7%, and 25.4% vs 22.2% respectively [20]. Also the mean serum glucose level was somewhat higher than that in the 2003–2005 WOBASZ study, where in women aged 20–34 and 35–44 years values were 4.4 mmol/l and 4.7 mmol/l respectively. Diabetes was found in 1.2% of our participants, compared to only 0.5% in the WOBASZ study performed a decade ago [15]. Over the last ten years, the hypertension rate in Polish females of childbearing age increased from about 6% to 15.7%. In summary, during the last decade, the rates of excessive body mass and the related metabolic disorders increased markedly in the population of Polish women of childbearing age. However, it is worth mentioning that an analysis of the WOBASZ data collected in years 2003 to 2005 showed that physical activity is an important protective factor against metabolic complications of overweight and obesity in women of childbearing age [23]. Thus, regular exercise should be encouraged in this population group.

As mentioned in the introduction, excessive body mass and the related metabolic disorders are a great threat to procreative health. They may impair fertility, and also foster development of such pregnancy complications as HDP and GDM. Besides, they may contribute to fetal macrosomia and an increased risk of diabetes, arterial hypertension, lipid disorders and cardiovascular disease in the child’s adulthood. It should be noted that prevalence of thyroid disease was also higher in older age groups. This is of concern because both hypo- and hyperthyroidism may impair fetal development and the course of pregnancy [24].

The trends for increased rates of many of the described disorders with age is worrying in the light of a tendency to postpone pregnancy, observed in Poland in the last decades,. The age range of the highest fertility rates has shifted since the nineties from the 20–24 year age group to the 25–29 year age group. Simultaneously, fertility rates among the 30–34 year age group have increased considerably. The median age of women giving birth to a child increased from 26 years in 2000 to somewhat above 29 years in 2013, and the age of mothers giving birth to their first child increased from before 24 years to slightly above 27 years [25].

This tendency to postpone a decision for parenthood, and the observed trend of increase in rates of excessive body mass and the related metabolic disorders with age, as well as the increase in the prevalence of these abnormalities in the last decade, may result in an increase in reproductive disorders. It seems that infertility is more prevalent now than it was several decades ago. It is estimated that infertility-related problems concern about 19% of couples in Poland [26]. There is no reliable data about the prevalence of pregnancy complications, such as GDM and HDP in Poland. It is estimated that gestational diabetes in various populations occur in 1%–14% of pregnancies [27]. It was shown recently that its prevalence among pregnant women in the US population increased from 0.3% in 1979–1980 to 5.8% in 2008–2010, which was related to the increase in maternal age and BMI, and was also linked to an increased rate of stillbirths [28]. Hypertensive disorders of pregnancy in the United States was observed in about 12% pregnancies [29]. It seems that the HDP rates are tending to increase. It was observed that the prevalence of HDP among US delivery hospitalizations increased from 67.2 per 1000 deliveries in 1998 to 81.4 per 1000 deliveries in 2006 [30]. It should be emphasized that GDM may lead to micro- or macrosomia in the newborn, whilst HDP may lead to intrauterine growth retardation. These pregnancy complications are also recognised risk factors for maternal cardiovascular disease [31].

Macrosomia in newborns is one of the complications observed in obese mothers. In Poland between 1992 and 2014, the percentage of newborns with high body mass (i.e. ≥4000 g) increased from 7.7% to 10.4% [32]. However, this data should be taken with caution, as the definition of a live birth was altered in 1994. Nonetheless, high body mass in newborns is common in Poland, leading to concerns about the risks of complications in later adult life, such as obesity, type 2 diabetes, arterial hypertension, and cardiovascular disease, as macrosomia is implicated in the intrauterine programming of these disorders [8, 9].

It should be emphasised that the epidemic of overweight and obesity which has been observed in recent decades, concerns not only women of childbearing age, but is a rising global phenomenon [33]. One of the causes may be the intrauterine programming of metabolic risk, which is of particular concern in pregnancies among obese and diabetic mothers. Therefore, gestational obesity and diabetes may largely contribute to the epidemic of obesity in the general population.

Unlike overweight and obesity, underweight is much less common in the Polish population of females of childbearing age. However, it is relatively common in the youngest age group. Being underweight also involves risk for pregnancy complications, especially for intrauterine growth retardation. Therefore, prevention efforts should not be focused exclusively on excessive body mass.

This study is limited by a relatively low response rate and also by a non-proportional sampling method. However, additional statistical analyses were performed that confirmed a similar age distribution between the general Polish population and the study group, thus indicating that the WOBASZ II study was indeed representative for the general Polish population.

## Conclusions

Overweight and obesity, especially of abdominal type, and related metabolic abnormalities are common in Polish women of childbearing age. Their prevalence tends to increase with age. Rates of these disorders increased in the last decade. In contrast to excessive body mass, underweight is the most common in the youngest age group and its prevalence tends to decrease with age. There is a need to monitor and treat abnormal body mass and metabolic disorders in Polish women of childbearing age.

## Abbreviations

AAMI: Association for the Advancement of Medical Instrumentation
ATP III: Adult Treatment Panel III
BMI: Body Mass Index
CDC: Centers for Disease Control and Prevention
DBP: Diastolic blood pressure
ESH/ESC: European Society of Cardiology and European Society of Hypertension
GDM: Gestational diabetes mellitus
HDL: High density lipoproteins
HDP: Hypertensive disorders of pregnancy
HTG: Hypertriglceridaemia
IDF: International Diabetes Federation
IFG: Impaired fasting glucose level
LDL: Low density lipoproteins
MS: Metabolic syndrome
NHANES: National Health and Nutrition Examination Survey
RIQAS: Random International Quality Assessment Scheme
SBP: Systolic blood pressure
TC: Total cholesterol
TG: Triglycerides
WHO: World Health Organization
WOBASZ II study: Multi-Centre National Population Health Examination Survey
